# Effect of mechanical power on intensive care mortality in ARDS patients

**DOI:** 10.1186/s13054-020-02963-x

**Published:** 2020-05-24

**Authors:** Silvia Coppola, Alessio Caccioppola, Sara Froio, Paolo Formenti, Valentina De Giorgis, Valentina Galanti, Dario Consonni, Davide Chiumello

**Affiliations:** 1 Department of Anesthesia and Intensive Care, ASST Santi Paolo e Carlo, San Paolo University Hospital, Milan, Italy; 2 Department of Health Sciences, University of Milan, Milan, Italy; 3 Epidemiology Unit, Fondazione IRCCS Ca’ Granda - Ospedale Maggiore Policlinico, Milan, Italy; 4 Coordinated Research Center on Respiratory Failure, University of Milan, Milan, Italy; 5 SC Anestesia e Rianimazione, ASST Santi Paolo e Carlo, Via Di Rudinì, Milan, Italy

**Keywords:** Mechanical power, Acute respiratory distress syndrome, Ventilator-induced lung injury, Intensive care mortality, Lung size, Compliance

## Abstract

**Background:**

In ARDS patients, mechanical ventilation should minimize ventilator-induced lung injury. The mechanical power which is the energy per unit time released to the respiratory system according to the applied tidal volume, PEEP, respiratory rate, and flow should reflect the ventilator-induced lung injury. However, similar levels of mechanical power applied in different lung sizes could be associated to different effects. The aim of this study was to assess the role both of the mechanical power and of the transpulmonary mechanical power, normalized to predicted body weight, respiratory system compliance, lung volume, and amount of aerated tissue on intensive care mortality.

**Methods:**

Retrospective analysis of ARDS patients previously enrolled in seven published studies. All patients were sedated, paralyzed, and mechanically ventilated.

After 20 min from a recruitment maneuver, partitioned respiratory mechanics measurements and blood gas analyses were performed with a PEEP of 5 cmH_2_O while the remaining setting was maintained unchanged from the baseline. A whole lung CT scan at 5 cmH_2_O of PEEP was performed to estimate the lung gas volume and the amount of well-inflated tissue.

Univariate and multivariable Poisson regression models with robust standard error were used to calculate risk ratios and 95% confidence intervals of ICU mortality.

**Results:**

Two hundred twenty-two ARDS patients were included; 88 (40%) died in ICU. Mechanical power was not different between survivors and non-survivors 14.97 [11.51–18.44] vs. 15.46 [12.33–21.45] J/min and did not affect intensive care mortality. The multivariable robust regression models showed that the mechanical power normalized to well-inflated tissue (RR 2.69 [95% CI 1.10–6.56], *p* = 0.029) and the mechanical power normalized to respiratory system compliance (RR 1.79 [95% CI 1.16–2.76], *p* = 0.008) were independently associated with intensive care mortality after adjusting for age, SAPS II, and ARDS severity. Also, transpulmonary mechanical power normalized to respiratory system compliance and to well-inflated tissue significantly increased intensive care mortality (RR 1.74 [1.11–2.70], *p* = 0.015; RR 3.01 [1.15–7.91], *p* = 0.025).

**Conclusions:**

In our ARDS population, there is not a causal relationship between the mechanical power itself and mortality, while mechanical power normalized to the compliance or to the amount of well-aerated tissue is independently associated to the intensive care mortality. Further studies are needed to confirm this data.

## Background

Acute respiratory distress syndrome (ARDS) is typically defined as an inflammatory pulmonary edema resulting from an acute damage of the alveoli [1, 2]. Mechanical ventilation, improving the severe hypoxemia and reducing the work of breathing, remains the cornerstone of ARDS management [3, 4]. However, ARDS is still accompanied with a mortality rate higher than 40% [5]. The mechanical forces generated during the mechanical ventilation by the interaction between the ventilator and the respiratory system can damage the lung, a process that has been called ventilator-induced lung injury (VILI) [6–8]. Previous randomized studies and retrospective data showed that excessive tidal volume, driving pressure, and positive end-expiratory pressure (PEEP) were related to a poor outcome [9–12]. Differently, lower respiratory rates and lower peak inspiratory pressures were associated with a decrease in-hospital mortality [13].

Up to now, all these factors have been evaluated separately [14], while the mechanical power, that is the amount of energy per unit of time generated by the mechanical ventilation and released on the respiratory system, unifying the mechanical drivers of VILI, has been proposed as a determinant of the VILI pathogenesis [15–17]. According to the classical equation of motion of the respiratory system, the energy applied to the respiratory system, per unit of time, depends on the mechanical properties of the lung (elastance and resistance), the applied tidal volume, the inspiratory flow, and the PEEP level [18]. For example, a reduction of the tidal volume together with an increase of the respiratory rate could increase or decrease the total energy delivered to the lung [16]. Experimental data, based on lung CT scan characteristics, suggested that a mechanical power higher of 12 J/min could generate VILI, irrespective of the different combinations of each component [17]. Thus, mechanical power should be superior to each of the individual components of the ventilator setting in modulating the final effect on the VILI [15]. However, no human data are available on the safe threshold of mechanical power for the developing of VILI.

A previous retrospective study enrolling 8207 critically ill mechanically ventilated patients reported that a mechanical power higher than 17 J/min, computed at the second day after ICU admission, was independently associated with higher hospital mortality [19]. However, similar values of mechanical power could result in different effects on the respiratory system, according both to the dimension of the ventilated lung (i.e., size of the baby lung) and to the relationship between lung and chest wall elastance (i.e., transpulmonary pressure) [16, 20–23]. In fact, for similar values of mechanical power, a higher or lower energy could be delivered respectively in case of a smaller or larger ventilated lung surface. Zhang et al., normalizing the mechanical power to the predicted body weight, as a surrogate of the lung size, reported that the normalized mechanical power better predicts in-hospital mortality [24].

The aim of this study was to investigate a possible role of mechanical power and transpulmonary mechanical power, adjusted for predicted body weight, respiratory system compliance, lung volume, and amount of aerated tissue on the intensive care mortality.

## Methods

### Study population

The study is a retrospective analysis of ARDS patients previously enrolled in seven published studies [2, 25–30]. The institutional review board of each hospital approved each study and written consent was obtained according to the regulations applied in each institution.

At baseline, patients were maintained deeply sedated and paralyzed, ventilated in volume control with a square wave form without any inspiratory pause, applying a tidal volume between 6 and 8 mL/kg of ideal body weight with a PEEP value set by the attending physician to ensure an arterial saturation between 93 and 97%. At baseline, data were collected including age, sex, body mass index, Simplified Acute Physiology Score (SAPS II), cause for ARDS, PaO_2_/FiO_2_, PaCO_2_, and clinical mechanical ventilator setting (PEEP, respiratory rate, tidal volume).

ARDS patients were classified as mild (200 mmHg < PaO_2_/FiO_2_ ratio ≤ 300 mmHg), moderate (100 mmHg < PaO_2_/FiO_2_ ratio ≤ 200 mmHg), or severe (PaO_2_/FiO_2_ ratio ≤ 100 mmHg) according to the Berlin definition [31].

### Mechanical ventilation setting

To standardize the lung volume history, a recruitment maneuver was performed in pressure-controlled ventilation at PEEP of 5 cmH_2_O, with a plateau pressure of 45 cmH_2_O, I:E 1:1, and respiratory rate of 10 breaths/min for 2 min [25]. After 20 min from the recruitment maneuver, all the respiratory mechanics measurements and blood gas analyses were performed with a PEEP of 5 cmH_2_O while the remaining setting was maintained unchanged from the baseline.

### CT scan acquisition and analysis

Patients were moved to the radiology department and a whole lung CT scan at 5 cmH_2_O of PEEP was performed after a recruitment maneuver. The lung gas volume and amount of well-inflated tissue were computed as previously described [32].

### Respiratory mechanics

Briefly, the respiratory flow rate was measured with a heated pneumotachograph (Fleisch n°2, Fleisch, Lausanne, Switzerland). Airway pressure was measured proximally to the endotracheal tube with a dedicated pressure transducer (MPX 2010 DP. Motorola, Solna, Sweden). Esophageal pressure was measured with a radio-opaque balloon (SmartCath Bicore, USA), positioned in the lower third of the esophagus, inflated with 1.0–1.5 mL of air and connected to a pressure transducer (MPX 2010 DP. Motorola, Solna, Sweden). All traces were sampled at 100 Hz and processed on a dedicated data acquisition system (Colligo and Computo, www.elekton.it) [33].

### Data analysis and calculation

All the data analysis was computed in intensive care survivors and not survivors.

#### Mechanical power

According to the equation of motion, the pressure inside the respiratory system is equal to the following:

1 $$ \mathrm{Pressure}={E}_{\left(\mathrm{RS}\right)}\times \mathrm{TV}+{R}_{\mathrm{AW}}\times F+\mathrm{PEEP} $$

where *E*_(RS)_ is the elastance of the respiratory system, *R*_aw_ is the total airway resistance, *F* is the inspiratory flow, and TV is the delivered tidal volume.

In passive patients (sedated and paralyzed), all the energy delivered to the respiratory system to increase the volume for a given minute ventilation (i.e., the mechanical power) is equal to the following:

2 $$ {\mathrm{MP}}_{\mathrm{RS}}=\left({\mathrm{TV}}^2\times {E}_{\left(\mathrm{RS}\right)}\times \frac{1}{2}+\mathrm{TV}\times {R}_{\mathrm{aw}}\times F+\mathrm{TV}\times \mathrm{PEEP}\right)\times \mathrm{RR}\times 0.098 $$

where MP_RS_ in the computed mechanical power applied to the respiratory system and RR is the respiratory rate [16].

The 3 components of the mechanical power are as follows:

3 $$ \mathrm{Elastance}-\mathrm{related}\ \mathrm{component}=\left({\mathrm{TV}}^2\times {E}_{\left(\mathrm{RS}\right)}\times \frac{1}{2}\right)\times \mathrm{RR}\times 0.098 $$

4 $$ \mathrm{PEEP}-\mathrm{related}\ \mathrm{component}=\left(\mathrm{TV}\times \mathrm{PEEP}\right)\times \mathrm{RR}\times 0.098 $$

5 $$ \mathrm{Resistance}-\mathrm{related}\ \mathrm{component}=\left(\ \mathrm{TV}\times {R}_{\mathrm{aw}}\times F\right)\times \mathrm{RR}\times 0.098 $$

The transpulmonary mechanical power was computed considering the lung elastance instead of respiratory system in Eq. (2).

The mechanical and transpulmonary mechanical power were normalized to the predicted body weight, lung gas volume, amount of well-aerated tissue, and respiratory system compliance.

**Fig. 1:**
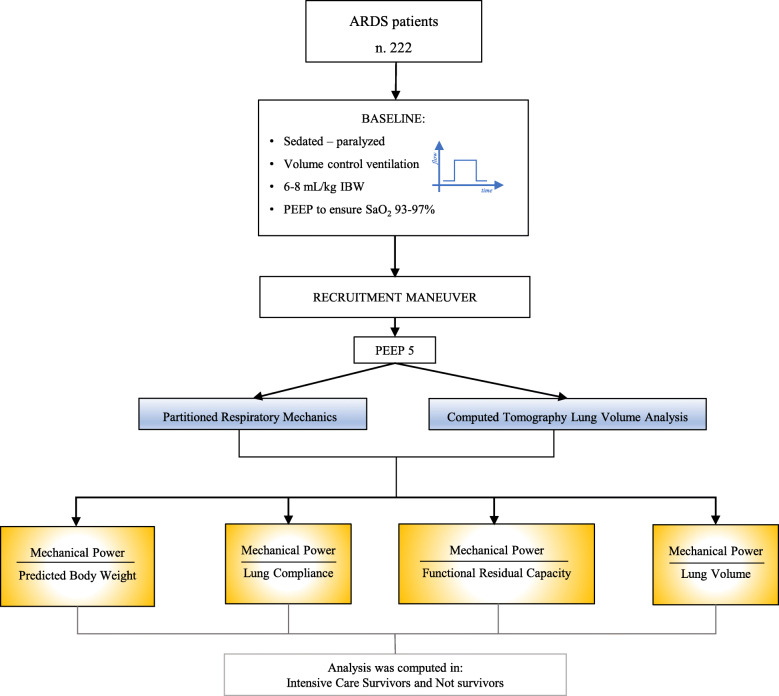
Flow chart of the study design

The driving pressure and partitioned respiratory mechanics (lung and chest wall) were computed according to the previously reported formula (see Additional file 1: Formulas of the physiological variables) (Fig. 1).

#### Statistical analysis

Quantitative variables are presented as mean (standard deviation, SD) or as median [interquartile range], respectively, for normal and non-normal distributions. To compare quantitative variables between survivors and ICU deaths, we used Student’s *t* test (for normally distributed data) or Wilcoxon-Mann-Whitney test. Chi-squared test was used for categorical data. We used univariate and multivariable Poisson regression models with robust standard error to calculate risk ratios (RR) of ICU mortality and 95% confidence intervals (CI) according to mechanical power and transpulmonary mechanical power. Multivariable models included age (years), SAPS II, and ARDS severity as adjustment covariates. Analyses were performed with Stata 15 (StataCorp. 2017) [34].

## Results

A total of 222 ARDS patients were included in the analysis; the median age was 61 [48–73] years and PaO_2_/FiO_2_ was 175 [133–224]. At enrollment, patients were ventilated with a clinical PEEP of 10 [10–12] cmH_2_O and with a tidal volume of predicted body weight of 7.8 [6.8–8.8] mL/kg.

One hundred and thirty-four patients survived (60%) and 88 (40%) patients died before intensive care discharge. Non-survivors, compared to survivors, were older, had higher SAPS II, and, at admission, received similar tidal volume/predicted body weight and level of PEEP, but had significantly lower PaO_2_/FiO_2_ and higher partial arterial pressure of carbon dioxide (Table 1).

**Table 1:** Baseline characteristics of the study population in relation to ICU mortality

	Survivors, *N* = 134	Non-survivors, *N* = 88	*p*
Age (years)	60 [43–70]	64 [54–75]	***0.005***
Female, *N* (%)	38 (31%)	31 (35%)	*0.48*
BMI (kg/m^2^)	24.85 [22.67–28.28]	24.25 (22.29–29.02)	*0.97*
ARDS category, *N* (%)			***0.001***
• Mild	32 (24)	9 (10)	
• Moderate	90 (67)	50 (57)	
• Severe	12 (9)	29 (33)	
Cause of ARDS, *N* (%)			***0.04***
• Pulmonary	76 (57)	37 (42)	
• Extrapulmonary	58 (43)	51 (58)	
PaO_2_/FiO_2_	195 [146–231]	153 [115.33–189.28]	***0.001***
PaCO_2_ (mmHg)	40 [35.92–4]	44.45 [39.15–52.9]	***0.001***
Respiratory rate (bpm)	16 (13–20)	17.5 (14–20)	*0.07*
Tidal volume (mL)	510 [450–600]	480 [420–550]	***0.004***
Tidal volume/body weight (mL/kg)	7.93 [7–9.1]	7.82 [6.7–8.47]	*0.12*
Clinical PEEP (cmH_2_O)	10 [10–12.5]	10 [10–12]	*0.68*
Driving pressure (cmH_2_O)	12.58 ± 3.31	14.03 ± 3.48	***0.004***
Respiratory system elastance (cmH_2_O/mL)	23.91 [18.44–29.15]	28.57 [22.97–34.87]	***0.001***
SAPS II	36 [29–47]	44 [37.75–56]	***0.001***
Intensive care unit stay (days)	19.5 [11.5–30.5]	15 [8–24]	***0.009***

Quantitative data are expressed as mean (standard deviation, SD) or median [interquartile range] as appropriate. Categorical data are presented as *N* (number of subjects) and percentages (%). Student’s *t* test or Wilcoxon-Mann-Whitney test, as appropriate, were used for continuous variable analysis, while chi-squared test were used for categorical variable analysis

*BMI* body mass index, *ARDS* acute respiratory distress syndrome, *SAPS* Simplified Acute Physiology Score

Italicized data are for statistically significant results

At 5 cmH_2_O of PEEP, tidal volume and respiratory rate were significantly lower and higher in non-survivors and survivors (480 [420–550] mL vs 510 [450–600], *p* = 0.004 and 28.57 [22.97–34.87] vs 23.91 [18.44–29.15] breath per minute, *p* = 0.001), the mechanical power was not different between survivors and non-survivors (14.97 [11.51–18.44] vs 15.46 [12.33–21.45] J/min, *p* = 0.300) (Table 1 and Table S2) and did not affect intensive care mortality, as shown from the univariate analysis (Table 2). Similarly, at the same PEEP, the two other components of mechanical power (respiratory system elastance and airway resistance) were not different between groups (see Additional file 2: Table S2). On the contrary, mechanical power normalized compliance and well-inflated tissue significantly increased ICU mortality with RR of 2.69 [0.75–9.57], *p* = 0.127; 1.88 [1.21–2.92], *p* = 0.005; and 3.60 [1.19–10.87], *p* = 0.023, respectively (Table 2). Mechanical power normalized to predicted body weight and to the lung gas volume did not increase ICU mortality (RR 2.69 [0.75–9.57], *p* = 0.127; and RR 1.01 [0.99–1.02, *p* = 0.167], respectively).

**Table 2:** Predictive performance of ventilatory variables for Intensive care unit mortality

Variables	RR	95% CI	*p*
Tidal volume (mL)	0.14	0.03–0.77	***0.024***
Airway plateau pressure (cmH_2_O)	1.04	1.00–1.08	***0.043***
Respiratory system elastance (cmH_2_O/mL)	1.03	1.01–1.04	***0.001***
Lung elastance (cmH_2_O/mL)	1.01	1.00–1.03	***0.025***
Chest wall elastance (cmH_2_O/mL)	1.00	0.93–0.96	*0.926*
MP (J/min)	1.01	0.99–1.03	*0.342*
MP_PBW (J/min/Kg)	2.69	0.75–9.57	*0.127*
MP_C_RS_ (J/min/mL/cmH_2_O)	1.88	1.21–2.92	***0.005***
MP_well-inflated tissue (J/min/g)	3.60	1.19–10.87	***0.023***
MP_lung gas volume (J/min/mL)	1.01	0.99–1.02	*0.167*
Transpulmonary MP_PBW (J/min/kg)	1.91	0.55–6.67	*0.305*
Transpulmonary MP_C_RS_ (J/min/mL/cmH_2_O)	1.01	0.99–1.02	*0.195*
Transpulmonary MP_well-inflated tissue (J/min/g)	3.91	1.12–13.61	***0.032***
Transpulmonary MP_lung total gas (J/min/mL)	1.01	0.99–1.01	*0.190*
Driving pressure (cmH_2_O)	1.04	1.00–1.08	***0.045***

Risk ratios (RR) and 95% confidence intervals (CI) of intensive care unit (ICU) mortality calculated with univariate Poisson regression with robust standard error

*MP* mechanical power, *PBW* predicted body weight, *C*_*RS*_ respiratory system compliance

Italicized data are for statistically significant results

In the univariate analysis, transpulmonary mechanical power did not influence intensive care mortality (see Additional file 3: Table S2) while transpulmonary mechanical power normalized to well-aerated tissue increased mortality with RR of 3.91 [1.12–13.61], *p* = 0.036 for one unit increase (Table 2).

### Risk factors for intensive care mortality

**Fig. 2:**
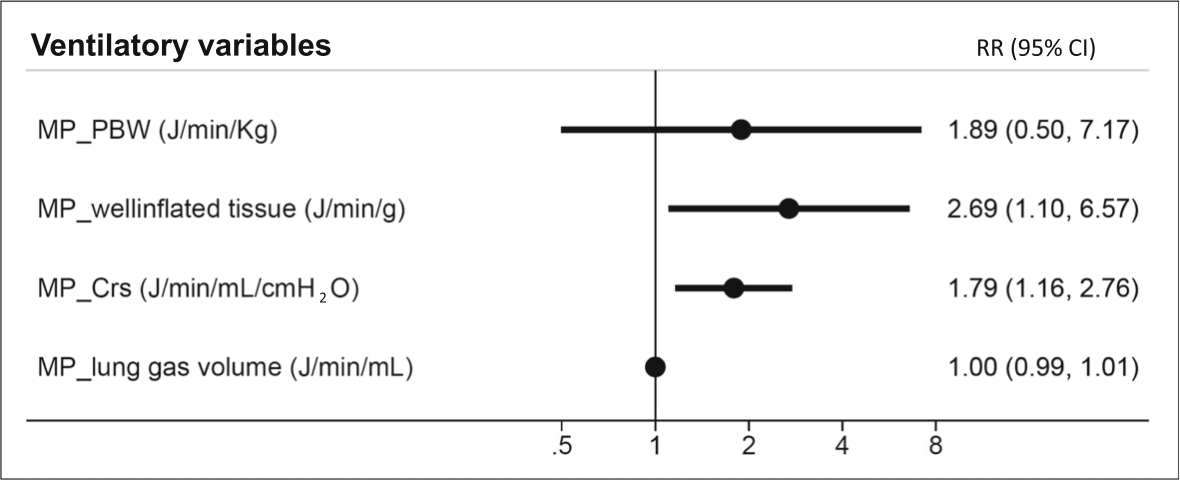
Predictive performance of ventilatory variables for Intensive care unit mortality. Relative risk (RR) and 95% confidence intervals (CI) of intensive care unit (ICU) mortality calculated with multivariable Poisson regression models with robust standard error

The mechanical power normalized to well-inflated tissue (RR 2.69 [95% CI 1.10–6.56]; *p* = 0.029) and the mechanical power normalized to respiratory system compliance (RR 1.79 [95% CI 1.16–2.76]; *p* = 0.008) remained independently associated with intensive care mortality after adjusting for age, SAPS II, and ARDS severity (Tables 3 and 4; Fig. 2).

**Table 3:** Multivariable regression models investigating risk factors for ICU mortality in ARDS patients

**ICU mortality**	**RR**	**95% CI**	***p***	**ICU mortality**	**RR**	**95% CI**	***p***
Age (with each 1-year increase)	1.01	1.00 - 1.02	0.038	Age (with each 1-year increase)	1.01	1.00 - 1.03	0.023
SAPS II (with each 1-point increase)	1.01	1.00 - 1.02	0.005	SAPS II (with each 1-point increase)	1.01	1.00 - 1.02	0.008
100 < PaO_2_ / FiO_2_ ≤ 200	1.41	0.71 - 2.82	0.325	100 < PaO_2_ / FiO_2_ ≤ 200	1.27	0.63 - 2.53	0.498
PaO_2_ / FiO_2_ ≤ 100	2.81	1.41 - 5.61	0.003	PaO_2_ / FiO_2_ ≤ 100	2.70	1.36 - 5.34	0.004
MP_PBW (J/min/Kg)	1.89	0.50 - 7.17	0.346	MP_well inflated tissue (J/min/g)	2.69	1.10 - 6.57	0.029
**ICU mortality**	**RR**	**95% CI**	***p***	**ICU mortality**	**RR**	**95% CI**	***p***
Age (with each 1-year increase)	1.01	1.00 - 1.02	0.022	Age (with each 1-year increase)	1.01	1.00 - 1.03	0.018
SAPS II (with each 1-point increase)	1.01	1.00 - 1.02	0.011	SAPS II (with each 1-point increase)	1.01	1.00 - 1.02	0.010
100 < PaO_2_ / FiO_2_ ≤ 200	1.38	0.69 - 2.75	0.359	100 < PaO_2_ / FiO_2_ ≤ 200	1.26	0.63 - 2.53	0.506
PaO_2_ / FiO_2_ ≤ 100	2.71	1.36 - 5.42	0.005	PaO_2_ / FiO_2_ ≤ 100	2.68	1.35 - 5.32	0.005
MP_CRS (J/min/mL/cmH2O)	1.79	1.16 - 2.76	0.008	MP_lung gas volume (J/min/mL)	1.00	0.99 - 1.01	0.177

Relative risk (RR) and 95% confidence intervals (CI) of intensive care unit (ICU) mortality calculated with multivariable Poisson regression models with robust standard error

Effects of age (years), SAPS II (reference: SAPS II simplified acute physiology score), ARDS severity (PaO_2_/FiO_2_; reference > 200), mechanical power normalized to predicted body weight (MP_PBW), mechanical power normalized to well inflated tissue (MP_well inflated tissue), mechanical power normalized to respiratory system compliance (MP_CRS), mechanical power normalized to lung gas volume (MP_lung gas volume)

**Table 4:** Multivariable regression models investigating risk factors for ICU mortality in ARDS patients

**ICU mortality**	**RR**	**95% CI**	***p***	**ICU mortality**	**RR**	**95% CI**	***p***
Age (with each 1-year increase)	1.01	1.00 - 1.02	0.041	Age (with each 1-year increase)	1.01	1.01- 1.02	0.024
SAPS II (with each 1-point increase)	1.01	1.00 - 1.02	0.004	SAPS II (with each 1-point increase)	1.01	1.01- 1.02	0.007
100 < PaO_2_ / FiO_2_ ≤ 200	1.47	0.74 - 2.94	0.274	100 < PaO_2_ / FiO_2_ ≤ 200	1.32	0.66- 2.64	0.438
PaO_2_ / FiO_2_ ≤ 100	2.95	1.48 - 5.89	0.002	PaO_2_ / FiO_2_ ≤ 100	2.80	1.40- 5.56	0.003
Transpulmonary MP_PBW (J/min/Kg)	1.68	0.48 - 5.93	0.419	Transpulmonary MP_well inflated tissue(J/min/g)	3.01	1.15- 7.91	0.025
**ICU mortality**	**RR**	**95% CI**	***p***	**ICU mortality**	**RR**	**95% CI**	***p***
Age (with each 1-year increase)	1.01	1.00 - 1.03	0.024	Age (with each 1-year increase)	1.01	1.00 - 1.03	0.019
SAPS II (with each 1-point increase)	1.01	1.00 - 1.02	0.008	SAPS II (with each 1-point increase)	1.01	1.00 - 1.02	0.009
100 < PaO_2_ / FiO_2_ ≤ 200	1.44	0.72 - 2.88	0.300	100 < PaO_2_ / FiO_2_ ≤ 200	1.31	0.65 - 2.63	0.444
PaO_2_ / FiO_2_ ≤ 100	2.85	1.42 - 5.70	0.003	PaO_2_ / FiO_2_ ≤ 100	2.78	1.39 - 5.54	0.004
Transpulmonary MP_C_RS_ (J/min/mL/cmH_2_O)	1.74	1.11 - 2.70	0.015	Transpulmonary MP_lung gas volume (J/min/g)	1.00	0.99 - 1.01	0.157

Relative risk (RR) and 95% confidence intervals (CI) of intensive care unit (ICU) mortality calculated with multivariable Poisson regression models with robust standard error

Effects of age (years), SAPS II (reference: SAPS II simplified acute physiology score), ARDS severity (PaO_2_/FiO_2_; reference > 200), transpulmonary mechanical power normalized to predicted body weight (Transpulmonary MP_PBW), transpulmonary mechanical power normalized to well inflated tissue (Transpulmonary MP_well inflated tissue), transpulmonary mechanical power normalized to respiratory system compliance (Transpulmonary MP_CRS), transpulmonary mechanical power normalized to lung gas volume (Transpulmonary MP_lung gas volume)

Considering the transpulmonary mechanical power in patients with similar age, SAPS II, and ARDS severity, the transpulmonary mechanical power normalized to well-inflated tissue significantly increased intensive care mortality (RR 3.01 [1.15–7.91]; *p* = 0.025), as well as the transpulmonary mechanical power normalized to the compliance (RR 1.74 [1.11–2.70]; *p* = 0.015) (Tables 3 and 4).

## Discussion

**Fig. 3:**
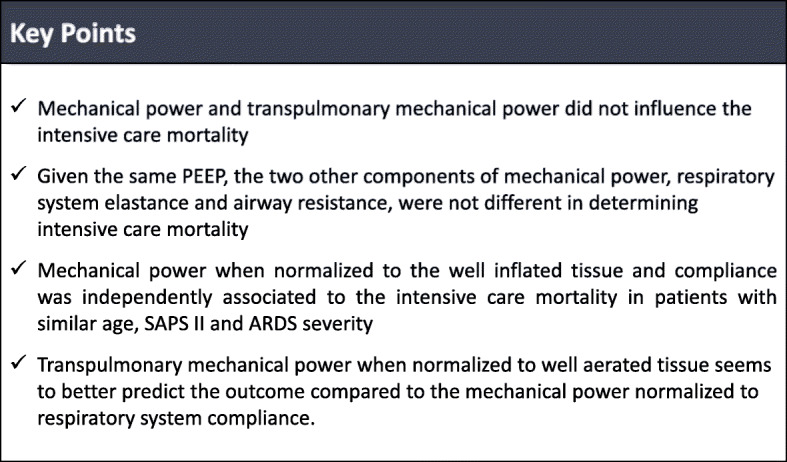
Key points

The results of the present study showed that (1) mechanical power and transpulmonary mechanical power did not influence the intensive care mortality; (2) given the same PEEP, the two other components of mechanical power, respiratory system elastance and airway resistance, were not different in determining the outcome; (3) the mechanical power when normalized to the well-inflated tissue and compliance was independently associated to the intensive care mortality in patients with similar age, SAPS II, and ARDS severity; and (4) the transpulmonary mechanical power when normalized to well-aerated tissue seems to better predict the outcome compared to the mechanical power normalized to respiratory system compliance (Fig. 3).

In ARDS, lung protective ventilation strategies should provide adequate gas exchange and minimize VILI [4, 35]. VILI has been mainly recognized to be associated to barotrauma (excessive pressure), volotrauma (excessive volume), and atelectrauma [14, 36]. Therefore, several strategies of mechanical ventilation focused on the decrease of each potential determinant of VILI, such as tidal volume, respiratory rate, and PEEP, have been proposed [9–12, 37, 38]. In this context, the reduction of mechanical power, that is the total energy released into the lung per unit time, derived from the interaction between the ventilator setting and lung conditions, could be considered an alternative approach to minimize VILI [18].

Originally, the mechanical power was measured as the product of airway pressure and tidal volume during each inflation times the respiratory rate [18]. In the present study, enrolling volume-controlled mechanically ventilated patients with a constant flow (square waveform), the mechanical power was mathematically computed according to the equation proposed by Gattinoni et al., which has been previously validated [16]. It is worth to remind that to estimate the real mechanical power, it is requested that patients should be well relaxed without any active inspiratory efforts. Our population included only sedated and paralyzed patients in whom the mechanical power was estimated at 5 cmH_2_O of PEEP. This low level of PEEP compared to higher levels has been reported to better describe the severity of the lung injury, stratify the risk, and predict the outcome [39, 40].

The mechanical power directly acting on the extracellular lung matrix modulates the VILI ranging from an inflammatory activation to a mechanical rupture of the lung [17]. Being the mechanical power originated by pressure, volume, and respiratory rate, the same mechanical power can be developed by a different combination of these factors.

Recent experimental data showed that when the mechanical power increased by a higher respiratory rate or level of PEEP, it was able to promote lung inflammation and edema and to reduce lung compliance [17, 41]. In a subsequent study of 1705 mechanically ventilated patients, the mechanical power was independently associated to a progression to ARDS [42]. To date, only few data are available in ARDS patients and a limited group of studies has assessed the relationship between the mechanical power and the survival [43–45] Among these, Parhar et al., including patients admitted from four intensive care units, reported that the mechanical power significantly increased with the severity of the ARDS and a mechanical power higher than 22 J/min was associated with a worse hospital and 3-year survival [43]. Similarly, Guerin et al., by applying an incomplete equation of the mechanical power, which did not take into account the resistive component, in a retrospective analysis of two previously published randomized controlled trials, found that mechanical power was associated to 90 days outcome [44]. On the contrary, although the mechanical power was significantly reduced during ECMO treatment, it was not related to the survival [45].

In fact, in ARDS patients, changes in airway pressure, due to the combination of lung and chest wall characteristics, could not adequately reflect the lung condition, which is better assessed by the transpulmonary pressure. The transpulmonary pressure is equal to the stress applied only to the lung. This implies that for a certain value of mechanical power (i.e., generated by an airway pressure), the resulting transpulmonary mechanical power can be higher or lower, according to the alterations in chest and lung mechanical characteristics [18]. For example, a higher transpulmonary mechanical power in the presence of a sicker lung and a lower transpulmonary pressure when the chest wall is impaired.

In the present study, both the mechanical power resulting from the airway pressure and from the transpulmonary pressure were assessed to better investigate the role of the mechanical power on intensive care mortality. However, both of them were not related to the outcome as well as the investigated components of mechanical power (resistive and elastic with the same PEEP) were not different, suggesting that in ARDS patients, they did not affect the outcome. A possible explanation of these results could be that the mechanical power if not normalized to the lung size does not reflect the real amount of energy dissipated into the lung (i.e., amount of the generated VILI). The smaller is the lung, the lower should be the mechanical power to minimize the VILI [46]. ARDS patients are characterized by a high and unpredictable variability of the lung size (i.e., baby lung) [37, 47]. Respiratory compliance was found well correlated with the amount of normally aerated tissue, reflecting the dimension of baby lung [21]**.** Thus, it has been proposed to normalize the mechanical power according to the predicted body weight, the compliance, or the amount of well-inflated tissue [14, 16, 48]. Zhang et al. found that the normalized mechanical power on the predicted body weight showed a better accuracy compared to the not normalized mechanical power in predicting the mortality [24]. Unfortunately, although the normalization to the predicted body weight is an easy method, in ARDS patients, the lung size is not proportional to the body weight [47]. Actually, we found that in patients with similar age, SAPS II, and ARDS severity, both the mechanical power normalized to compliance and to well-inflated tissue independently increased the intensive care mortality of 1.78 and 2.64 times for one unit increase, respectively. Differently, the mechanical power normalized to the lung gas volume did not influence the outcome, probably because the lung gas volume does not reflect the amount of tissue in which the energy is dissipated in the lung without increasing the VILI. Our hypothesis is that the respiratory system compliance and the amount of well-inflated tissue should better reflect the amount of lung-aerated tissue exposed to the energy load during the mechanical ventilation. Furthermore, the transpulmonary mechanical power normalized to well-inflated tissue better predicted the mortality, suggesting that in addition to the amount of resistive capacity of the lung, assessed as compliance or amount of well-aerated tissue, the partitioned lung mechanics characteristics computed by esophageal pressure have a determinant role on the effect of mechanical power.

In the present study, in addition to the normalized mechanical power, also the driving pressure which is the tidal volume normalized to the compliance of respiratory system, it was associated to the intensive care mortality, both in the univariate analysis and in the multivariate analysis, (see Additional file 4: Table S3), confirming previous studies in ARDS patients in which the driving pressure has been independently related to the hospital outcome [9].

### Limitations

The present study has several limitations. First, the mechanical power was computed just one time and not during the intensive care stay; thus, it did not reflect the temporal changes of mechanical power really applied to the patients. Second, only mechanically ventilated patients during volume control ventilation with a constant flow were enrolled due to the inaccuracy of computing mechanical power in assisted ventilation or pressure control ventilation. Third, the mechanical power was normalized only considering indexes reflecting static conditions (compliance, lung size, and amount of well-inflated tissue). However, other lung conditions such as the degree of lung inhomogeneity and different pulmonary vascular pressure could modulate the risk of VILI independently of a higher or lower mechanical power [23, 49].

## Conclusions

In conclusion, the present results suggest that in our ARDS population, there is not a causal relationship between the mechanical power itself and mortality, while mechanical power normalized to the compliance or to the amount of well-aerated tissue is independently associated to the intensive care mortality. Further studies are needed to confirm this data.

## Supplementary information

**Additional file 1.** Formulas of the physiological variables. Formulas used in the paper are reported in the supplemental material.

**Additional file 2: Table S1.** Predictive performance of ventilatory variables for Intensive Care Unit mortality. Risk ratios (RR) and 95% confidence intervals (CI) of Intensive Care mortality calculated with univariate Poisson regression with robust standard error. MP mechanical power.

**Additional file 3: Table S2.** Values of mechanical power and transpulmonary mechanical power with stratification by Intensive care unit mortality. Results are expressed as mean (standard deviation) or median [I.Q. range] as appropriate. Student’s t or Mann-Whitney rank-sum tests were used for comparisons. *p* < 0.05. MP mechanical power; MP_ PBW Mechanical power normalized to predicted body weight; MP_C_RS_ Mechanical power normalized to respiratory system compliance.

**Additional file 4: Table S3.** Multivariable regression models investigating risk factors for ICU mortality in ARDS patients. Relative risk (RR) and 95% confidence intervals (CI) of intensive care unit (ICU) mortality calculated with multivariable Poisson regression models with robust standard error. Effects of age (years), SAPS II (reference: SAPS II simplified acute physiology score), ARDS severity (PaO_2_ / FiO_2_; reference > 200), mechanical power (MP), Driving Pressure.

## Data Availability

The datasets used and analyzed in the study are available from the corresponding author on reasonable request.

## Abbreviations

ARDS: Acute respiratory distress syndrome
CT: Computed tomography
PEEP: Positive end-expiratory pressure
VILI: Ventilator-induced lung injury
SAPS: II Simplified Acute Physiology Score
MP: Mechanical power
RR: Risk ratio
SD: Standard deviation
CI: Confidence interval
PBW: Predicted body weight

## References

[1] Ranieri VM, Rubenfeld GD, Thompson BT, Ferguson ND, Caldwell E, ARDS Definition Task Force (2012). Acute respiratory distress syndrome: the Berlin Definition. JAMA..

[2] Cressoni M, Chiumello D, Chiurazzi C, Brioni M, Algieri I, Gotti M (2016). Lung inhomogeneities, inflation and [18F]2-fluoro-2-deoxy-D-glucose uptake rate in acute respiratory distress syndrome. Eur Respir J.

[3] Fan E, Del Sorbo L, Goligher EC, Hodgson CL, Munshi L, Walkey AJ, et al. An official American Thoracic Society/European Society of Intensive Care Medicine/Society of Critical Care Medicine clinical practice guideline: mechanical ventilation in adult patients with acute respiratory distress syndrome [published correction appears in am J Respir Crit care med. 2017 Jun 1;195(11):1540]. Am J Respir Crit Care Med 2017;195(9):1253–1263. 10.1164/rccm.201703-0548ST 28459336

[4] Chiumello D, Brochard L, Marini JJ, Slutsky AS, Mancebo J, Ranieri VM (2017). Respiratory support in patients with acute respiratory distress syndrome: an expert opinion. Crit Care.

[5] Chiumello D, Coppola S, Froio S, Gotti M (2016). What’s next after ARDS: long-term outcomes. Respir Care.

[6] Slutsky AS, Ranieri VM (2014). Ventilator-induced lung injury. N Engl J Med.

[7] Gattinoni L, Carlesso E, Cadringher P, Valenza F, Vagginelli F, Chiumello D (2003). Physical and biological triggers of ventilator-induced lung injury and its prevention. Eur Respir J Suppl..

[8] Chiumello D, Pristine G, Slutsky AS (1999). Mechanical ventilation affects local and systemic cytokines in an animal model of acute respiratory distress syndrome. Am J Respir Crit Care Med.

[9] Amato MB, Meade MO, Slutsky AS, Brochard L, Costa EL, Schoenfeld DA (2015). Driving pressure and survival in the acute respiratory distress syndrome. N Engl J Med.

[10] Brower RG, Matthay MA, Morris A, Schoenfeld D, Thompson BT, Acute Respiratory Distress Syndrome Network (2000). Ventilation with lower tidal volumes as compared with traditional tidal volumes for acute lung injury and the acute respiratory distress syndrome. N Engl J Med.

[11] Putensen C, Theuerkauf N, Zinserling J, Wrigge H, Pelosi P (2009). Meta-analysis: ventilation strategies and outcomes of the acute respiratory distress syndrome and acute lung injury. Ann Intern Med..

[12] Meade MO, Cook DJ, Guyatt GH, Slutsky AS, Arabi YM, Cooper DJ (2008). Ventilation strategy using low tidal volumes, recruitment maneuvers, and high positive end-expiratory pressure for acute lung injury and acute respiratory distress syndrome: a randomized controlled trial. JAMA..

[13] Laffey JG, Bellani G, Pham T, Fan E, Madotto F, Bajwa EK, et al. Potentially modifiable factors contributing to outcome from acute respiratory distress syndrome: the LUNG SAFE study [published correction appears in Intensive Care Med. 2017 Nov 14;]. Intensive Care Med 2016;42(12):1865–1876. 10.1007/s00134-016-4571-5 27757516

[14] Tonetti T, Vasques F, Rapetti F, Maiolo G, Collino F, Romitti F (2017). Driving pressure and mechanical power: new targets for VILI prevention. Ann Transl Med.

[15] Marini John J., Rocco Patricia R. M., Gattinoni Luciano (2020). Static and Dynamic Contributors to Ventilator-induced Lung Injury in Clinical Practice. Pressure, Energy, and Power. American Journal of Respiratory and Critical Care Medicine.

[16] Gattinoni L, Tonetti T, Cressoni M, Cadringher P, Herrmann P, Moerer O (2016). Ventilator-related causes of lung injury: the mechanical power. Intensive Care Med.

[17] Cressoni M, Gotti M, Chiurazzi C, Massari D, Algieri I, Amini M (2016). Mechanical power and development of ventilator-induced lung injury. Anesthesiology..

[18] Marini JJ, Rodriguez RM, Lamb V (1986). Bedside estimation of the inspiratory work of breathing during mechanical ventilation. Chest..

[19] Serpa Neto A, Deliberato RO, Johnson AEW, Bos LD, Amorim P, Pereira SM (2018). Mechanical power of ventilation is associated with mortality in critically ill patients: an analysis of patients in two observational cohorts. Intensive Care Med.

[20] Chiumello D, Carlesso E, Cadringher P, Caironi P, Valenza F, Polli F (2008). Lung stress and strain during mechanical ventilation for acute respiratory distress syndrome. Am J Respir Crit Care Med.

[21] Gattinoni L, Pesenti A (2005). The concept of “baby lung”. Intensive Care Med.

[22] Vasques F, Duscio E, Cipulli F, Romitti F, Quintel M, Gattinoni L (2018). Determinants and prevention of ventilator-induced lung injury. Crit Care Clin.

[23] Marini JJ, Jaber S (2016). Dynamic predictors of VILI risk: beyond the driving pressure. Intensive Care Med.

[24] Zhang Z, Zheng B, Liu N, Ge H, Hong Y (2019). Mechanical power normalized to predicted body weight as a predictor of mortality in patients with acute respiratory distress syndrome. Intensive Care Med.

[25] Gattinoni L, Caironi P, Cressoni M, Chiumello D, Ranieri VM, Quintel M (2006). Lung recruitment in patients with the acute respiratory distress syndrome. N Engl J Med.

[26] Cressoni M, Chiumello D, Algieri I, Brioni M, Chiurazzi C, Colombo A (2017). Opening pressures and atelectrauma in acute respiratory distress syndrome. Intensive Care Med.

[27] Chiumello D, Cressoni M, Carlesso E, Caspani ML, Marino A, Gallazzi E (2014). Bedside selection of positive end-expiratory pressure in mild, moderate, and severe acute respiratory distress syndrome. Crit Care Med.

[28] Chiumello D, Marino A, Brioni M, Cigada I, Menga F, Colombo A (2016). Lung recruitment assessed by respiratory mechanics and computed tomography in patients with acute respiratory distress syndrome. What is the relationship?. Am J Respir Crit Care Med.

[29] Chiumello D, Mongodi S, Algieri I, Vergani GL, Orlando A, Via G (2018). Assessment of lung aeration and recruitment by CT scan and ultrasound in acute respiratory distress syndrome patients. Crit Care Med.

[30] Chiumello D, Marino A, Cressoni M, Mietto C, Berto V, Gallazzi E (2013). Pleural effusion in patients with acute lung injury: a CT scan study. Crit Care Med.

[31] Ferguson ND, Fan E, Camporota L, Antonelli M, Anzueto A, Beale R (2012). The Berlin definition of ARDS: an expanded rationale, justification, and supplementary material. Intensive Care Med.

[32] Gattinoni L, Caironi P, Pelosi P, Goodman LR (2001). What has computed tomography taught us about the acute respiratory distress syndrome?. Am J Respir Crit Care Med.

[33] Chiumello D, Consonni D, Coppola S, Froio S, Crimella F, Colombo A (2016). The occlusion tests and end-expiratory esophageal pressure: measurements and comparison in controlled and assisted ventilation. Ann Intensive Care.

[34] Zou G (2004). A modified poisson regression approach to prospective studies with binary data. Am J Epidemiol.

[35] Coppola S, Caccioppola A, Froio S, Ferrari E, Gotti M, Formenti P (2019). Dynamic hyperinflation and intrinsic positive end-expiratory pressure in ARDS patients. Crit Care.

[36] Albaiceta GM, Blanch L (2011). Beyond volutrauma in ARDS: the critical role of lung tissue deformation. Crit Care.

[37] Gattinoni L, Marini JJ, Pesenti A, Quintel M, Mancebo J, Brochard L (2016). The “baby lung” became an adult. Intensive Care Med.

[38] Curley GF, Laffey JG, Zhang H, Slutsky AS (2016). Biotrauma and ventilator-induced lung injury: clinical implications. Chest..

[39] Caironi P, Carlesso E, Cressoni M, Chiumello D, Moerer O, Chiurazzi C (2015). Lung recruitability is better estimated according to the Berlin definition of acute respiratory distress syndrome at standard 5 cm H2O rather than higher positive end-expiratory pressure: a retrospective cohort study. Crit Care Med.

[40] Maiolo G, Collino F, Vasques F, Rapetti F, Tonetti T, Romitti F (2018). Reclassifying acute respiratory distress syndrome. Am J Respir Crit Care Med.

[41] Collino F, Rapetti F, Vasques F, Maiolo G, Tonetti T, Romitti F (2019). Positive end-expiratory pressure and mechanical power. Anesthesiology..

[42] Fuller BM, Page D, Stephens RJ, Roberts BW, Drewry AM, Ablordeppey E (2018). Pulmonary mechanics and mortality in mechanically ventilated patients without acute respiratory distress syndrome: a cohort study. Shock..

[43] Parhar KKS, Zjadewicz K, Soo A, Sutton A, Zjadewicz M, Doig L (2019). Epidemiology, mechanical power, and 3-year outcomes in acute respiratory distress syndrome patients using standardized screening. An observational cohort study. Ann Am Thorac Soc.

[44] Guérin C, Papazian L, Reignier J, Ayzac L, Loundou A, Forel JM (2016). Effect of driving pressure on mortality in ARDS patients during lung protective mechanical ventilation in two randomized controlled trials. Crit Care.

[45] Schmidt M, Pham T, Arcadipane A, Agerstrand C, Ohshimo S, Pellegrino V (2019). Mechanical ventilation management during extracorporeal membrane oxygenation for acute respiratory distress syndrome. An international multicenter prospective cohort. Am J Respir Crit Care Med.

[46] Silva PL, Ball L, Rocco PRM, Pelosi P (2019). Power to mechanical power to minimize ventilator-induced lung injury?. Intensive Care Med Exp.

[47] Chiumello D, Carlesso E, Brioni M, Cressoni M (2016). Airway driving pressure and lung stress in ARDS patients. Crit Care.

[48] Gattinoni L, Marini JJ, Collino F, Maiolo G, Rapetti F, Tonetti T (2017). The future of mechanical ventilation: lessons from the present and the past. Crit Care.

[49] Gattinoni L, Quintel M (2016). How ARDS should be treated. Crit Care.

