# Supplementary material for: Effect of mechanical power on intensive care mortality in ARDS patients
Source: Crit Care. 2020 May 24;24:246. doi: 10.1186/s13054-020-02963-x (PMC7245621; doi:10.1186/s13054-020-02963-x)
Supplement: Supplementary file 1 — Additional file 1. Formulas of the physiological variables. Formulas used in the paper are reported in the supplemental material. [file 13054_2020_2963_MOESM1_ESM.docx]

**Formulas of the physiological variables**

MP (Joule/min) = TV * RR * 0.098 * [P_Peak_ - 0.5 * (P_Peak_ - P_Plateau_)]

where: TV is the delivered Tidal Volume, RR the respiratory rate, P_Peak_ is the Peak pressure, P_Plateau_ is the Plateau pressure

MP_PBW (Joule/min/Kg) = MP / PBW

where predicted body weight was calculated as follows:

Males = 50 + 0.91 *(height *100 -152.4)

Females = 45.5 + 0.91 *(height *100 -152.4)

MP_well inflated tissue (Joule/min/grams) = MP_RS_ / well inflated tissue

MP_lung gas volume (Joule/min/mL) = MP_RS_ / (lung gas volume / 1000)

MP_Compliance (Joule/min/mL/cmH_2_O) = MP_RS_ / [Vt / (P_Peak_ - P_Plateau_)]

MP_Elastance_ (Joule/min) = (TV^2^ * E_RS_ / 2) * RR * 0.098

where: E_RS_ is the elastance of the respiratory system, RR the respiratory rate

MP_PEEP_ (Joule/min) = (PEEP * TV) * RR * 0.098

where PEEP is the positive end expiratory pressure, RR the respiratory rate

MP_Resistance_ (Joule/min) = (TV * F * Raw) * RR * 0.098

where Raw is the total airway resistance, F is the inspiratory flow, RR the respiratory rate

Transpulmonary MP = TV^2^ * E_L_ / 2 + TV * Raw * F + TV * PEEP * E_L_ / E_RS_ * RR * 0.098

where E_L_ is the lung elastance, Raw is the total airway resistance, F is the inspiratory flow, PEEP is the positive end expiratory pressure

Transpulmonary MP _elastance_ (Joule/min) = (TV^2^ * E_L_ / 2) * 0.098 * RR

where E_L_ is the lung elastance

Transpulmonary MP _Resistance_ (Joule/min) = Transpulmonary MP / Raw

where Raw is the total airway resistance

Transpulmonary MP_PEEP_ (Joule/min) = (TV * PEEP * E_L_ / 2) * RR * 0.098

where E_L_ is the lung elastance

Driving pressure (cmH_2_O) = P_Plateau_ – PEEP

Respiratory system Elastance (cmH_2_O*mL^-1^) = P_Plateau_ – PEEP / TV

Chest wall Elastance (cmH_2_O*mL^-1^) = (End-inspiratory esophageal pressure – End-expiratory esophageal pressure) / TV

Lung Elastance (cmH_2_O*mL^-1^) = (Respiratory system elastance - Chest wall elastance) / TV

Total airway Resistance (cmH_2_O/L/sec) = (P_Peak_ - P_Plateau_) / F

where F is the inspiratory flow
