# Supplementary material for: Effect of mechanical power on intensive care mortality in ARDS patients
Source: Crit Care. 2020 May 24;24:246. doi: 10.1186/s13054-020-02963-x (PMC7245621; doi:10.1186/s13054-020-02963-x)
Supplement: Supplementary file 2 — Additional file 2: Table S1. Predictive performance of ventilatory variables for Intensive Care Unit mortality. Risk ratios (RR) and 95% confidence intervals (CI) of Intensive Care mortality calculated with univariate Poisson regression with robust standard error. MP mechanical power. [file 13054_2020_2963_MOESM2_ESM.docx]

**Table S1.** Predictive performance of ventilatory variables for Intensive Care Unit mortality.

| Variables | RR | I.C. 95% | *p* |
| --- | --- | --- | --- |
| PaO_2_/FiO_2_ ratio | 0.99 | 0.99-1.00 | ***0.004*** |
| MP (J/min) | 1.01 | 0.99-1.03 | *0.342* |
| MP elastance (J/min) | 1.05 | 0.99-1.12 | 0.112 |
| MP resistance (J/min/cmH_2_O/L/sec) | 1.01 | 0.97-1.06 | 0.728 |
| Transpulmonary MP (J/min) | 1.00 | 0.98-1.04 | 0.703 |

Risk ratios (RR) and 95% confidence intervals (CI) of Intensive Care mortality calculated with univariate Poisson regression with robust standard error.

MP mechanical power.
