# Supplementary material for: Effect of mechanical power on intensive care mortality in ARDS patients
Source: Crit Care. 2020 May 24;24:246. doi: 10.1186/s13054-020-02963-x (PMC7245621; doi:10.1186/s13054-020-02963-x)
Supplement: Supplementary file 3 — Additional file 3: Table S2. Values of mechanical power and transpulmonary mechanical power with stratification by Intensive care unit mortality. Results are expressed as mean (standard deviation) or median [I.Q. range] as appropriate. Student’s t or Mann-Whitney rank-sum tests were used for comparisons. p < 0.05. MP mechanical power; MP_ PBW Mechanical power normalized to predicted body weight; MP_CRS Mechanical power normalized to respiratory system compliance. [file 13054_2020_2963_MOESM3_ESM.docx]

**Table S2.** Values of mechanical power and transpulmonary mechanical power with stratification by Intensive care unit mortality.

|  | All population | Survivors | Non survivors | *p* |
| --- | --- | --- | --- | --- |
| MP (J/min) | 15.23 [11.77-20.22] | 14.97 [11.51-18.44] | 15.46 [12.33-21.45] | **0.300** |
| MP_PBW (J/min/Kg) | 0.23 [0.19-0.32] | 0.22 [0.18-0.31] | 0.24 [0.19-0.33] | 0.079 |
| MP_C_RS_ (J/min/mL/cmH_2_O) | 0.36 [0.27-0.55] | 0.346 [0.24-0.50] | 0.38 [0.28-0.62] | **0.059** |
| MP_lung gas volume (J/min/L) | 15.24 [9.88-23.42] | 13.76 [9.35-20.96] | 17.22[11.38-25.51] | **0.033** |
| MP_well inflated tissue (J/min/g) | 0.04 [0.03-0.07] | 0.04 [0.03-0.06] | 0.04 [0.03-0.07] | **0.023** |
| MP elastance (J/min) | 5.17 [3.91-7.19] | 4.89 [3.90-6.74] | 5.24 [3.92-7.72] | 0.217 |
| MP resistance (J/min/cmH_2_O/L/sec) | 5.61 [3.51 .8.07] | 5.54 [3.42-8.14] | 6.24 [3.92-1.99] | 0.345 |
| Transpulmonary MP (J/min) | 13.15 [10.10-18.16] | 13.51 [9.99-17.76] | 13.09 [10.42-19.3] | 0.658 |
| Transpulmonary MP_PBW (J/min/Kg) | 0.20 [0.16-0.30] | 0.20 [0.14-0.27] | 0.20 [0.17-0.30] | 0.326 |
| Transpulmonary MP_C_RS_ (J/min/mL/cmH_2_O) | 0.33 [0.23-0.48] | 0.31 [0.21-0.45] | 0.35 [0.24-0.58] | 0.119 |
| Transpulmonary MP_lung gas volume (J/min/L) | 12.97 [8.39-20.79] | 12.21 [7.78-19.22] | 14.95 [9.09-21.91] | 0.076 |
| Transpulmonary MP_well inflated tissue (J/min/g) | 0.040 [0.03 -0.06] | 0.04 [0.02-0.05] | 0.04 [0.03-0.07] | 0.060 |

Results are expressed as mean (standard deviation) or median [I.Q. range] as appropriate. Student’s t or Mann-Whitney rank-sum tests were used for comparisons. p < 0.05.

MP mechanical power; MP_ PBW Mechanical power normalized to predicted body weight; MP_C_RS_ Mechanical power normalized to respiratory system compliance.
