# Supplementary material for: Effect of mechanical power on intensive care mortality in ARDS patients
Source: Crit Care. 2020 May 24;24:246. doi: 10.1186/s13054-020-02963-x (PMC7245621; doi:10.1186/s13054-020-02963-x)
Supplement: Supplementary file 4 — Additional file 4: Table S3. Multivariable regression models investigating risk factors for ICU mortality in ARDS patients. Relative risk (RR) and 95% confidence intervals (CI) of intensive care unit (ICU) mortality calculated with multivariable Poisson regression models with robust standard error. Effects of age (years), SAPS II (reference: SAPS II simplified acute physiology score), ARDS severity (PaO2 / FiO2; reference > 200), mechanical power (MP), Driving Pressure. [file 13054_2020_2963_MOESM4_ESM.docx]

**Table S3.** Multivariable regression models investigating risk factors for ICU mortality in ARDS patients

| ICU mortality | RR | 95% CI | p |
| --- | --- | --- | --- |
| Age (with each 1-year increase) | 1.01 | 1.01 - 1.03 | 0.048 |
| SAPS II (with each 1-point increase) | 1.01 | 1.01 - 1.02 | 0.005 |
| 100< paO_2_/FiO_2_ ≤ 200 | 1.47 | 0.75 - 3.1 | 0.267 |
| paO_2_/FiO_2_ ≤ 100 | 3.02 | 1.51 - 6.01 | 0.002 |
| MP (J/min) | 1.00 | 0.98 - 1.02 | 0.862 |

| ICU mortality | RR | 95% CI | p |
| --- | --- | --- | --- |
| Age (with each 1-year increase) | 1.01 | 1.00 - 1.02 | 0.014 |
| SAPS II (with each 1-point increase) | 1.01 | 1.00 - 1.02 | 0.011 |
| 100 < paO_2_/FiO_2_ ≤ 200 | 1.53 | 0.76 - 3.08 | 0.232 |
| paO_2_/FiO_2_ ≤ 100 | 3.10 | 1.54 - 6.24 | 0.002 |
| Driving Pressure | 1.04 | 1.00 – 1.08 | 0.025 |

| ICU mortality | RR | 95% CI | p |
| --- | --- | --- | --- |
| Age (with each 1-year increase) | 1.01 | 1.01 - 1.02 | 0.016 |
| SAPS II (with each 1-point increase) | 1.01 | 1.01 - 1.3 | 0.009 |
| 100 < paO_2_/FiO_2_ ≤ 200 | 1.46 | 0.75 - 2.96 | 0.276 |
| paO_2_/FiO_2_ ≤100 | 3.06 | 1.53 - 6.09 | 0.001 |
| MP (J/min) | 0.99 | 0.96- 1.01 | 0.411 |
| Driving Pressure | 1.05 | 1.00-1.09 | 0.018 |

Relative risk (RR) and 95% confidence intervals (CI) of intensive care unit (ICU) mortality calculated with multivariable Poisson regression models with robust standard error.

Effects of age (years), SAPS II (reference: SAPS II simplified acute physiology score), ARDS severity (PaO_2_ / FiO_2_; reference > 200), mechanical power (MP), Driving Pressure.
